# Frequency of Infant Stroking Reported by Mothers Moderates the Effect of Prenatal Depression on Infant Behavioural and Physiological Outcomes

**DOI:** 10.1371/journal.pone.0045446

**Published:** 2012-10-16

**Authors:** Helen Sharp, Andrew Pickles, Michael Meaney, Kate Marshall, Florin Tibu, Jonathan Hill

**Affiliations:** 1 Institute of Psychology, Health and Society, University of Liverpool, Liverpool, United Kingdom; 2 Department of Biostatistics, Institute of Psychiatry, King's College London, London, United Kingdom; 3 Douglas Mental Health University Institute, McGill University, Montreal, Canada; 4 Mental Health and Neurodegeneration Research Group, University of Manchester, Manchester, United Kingdom; University of Western Brittany, France

## Abstract

Animal studies find that prenatal stress is associated with increased physiological and emotional reactivity later in life, mediated via fetal programming of the HPA axis through decreased glucocorticoid receptor (GR) gene expression. Post-natal behaviours, notably licking and grooming in rats, cause decreased behavioural indices of fear and reduced HPA axis reactivity mediated via increased GR gene expression. Post-natal maternal behaviours may therefore be expected to modify prenatal effects, but this has not previously been examined in humans. We examined whether, according to self-report, maternal stroking over the first weeks of life modified associations between prenatal depression and physiological and behavioral outcomes in infancy, hence mimicking effects of rodent licking and grooming. From a general population sample of 1233 first time mothers recruited at 20 weeks gestation we drew a stratified random sample of 316 for assessment at 32 weeks based on reported inter-partner psychological abuse, a risk to child development. Of these 271 provided data at 5, 9 and 29 weeks post delivery. Mothers reported how often they stroked their babies at 5 and 9 weeks. At 29 weeks vagal withdrawal to a stressor, a measure of physiological adaptability, and maternal reported negative emotionality were assessed. There was a significant interaction between prenatal depression and maternal stroking in the prediction of vagal reactivity to a stressor (p = .01), and maternal reports of infant anger proneness (p = .007) and fear (p = .043). Increasing maternal depression was associated with decreasing physiological adaptability, and with increasing negative emotionality, only in the presence of low maternal stroking. These initial findings in humans indicate that maternal stroking in infancy, as reported by mothers, has effects strongly resembling the effects of observed maternal behaviours in animals, pointing to future studies of the epigenetic, physiological and behavioral effects of maternal stroking.

## Introduction

Studies of animals and humans demonstrate sustained effects of prenatal stress on physiological stress reactivity and behavior in the offspring. In animals, these effects are modifiable by postnatal experience, notably in rats by early maternal tactile stimulation [1]–[5]. We report the first investigation in humans of moderation of prenatal stress effects by early human tactile stimulation on behavioural and physiological stress reactivity in infancy.

Prenatally stressed rats show evidence of increased fear and depression-like behaviors, and altered hypothalamic-pituitary-adrenal (HPA) and cardiovascular regulation [6]. These effects have been shown to be mediated via fetal programming of the HPA axis through decreased glucocorticoid receptor (GR) gene expression, resulting in impairments in feedback regulation of the HPA axis and elevated corticotrophin releasing factor (CRF) secretion in the hypothalamus [7]. CRF has multiple effects via receptors in the amydgala, hippocampus and brain stem which account for many of the effects of prenatal stress on cardiovascular and emotional reactivity. Consistent with the animal literature, human studies find that indices of prenatal stress such as maternal depression or anxiety in pregnancy also predict altered HPA reactivity [8], cardiovascular regulation [9],[10] and negative emotionality in infants [11], and conduct disorders, and emotional problems in children [1], [2], [12]. In this study we assessed physiological and behavioural outcomes in infancy likely to be influenced by prenatal stress via fetal programming of the HPA axis. The physiological measure was respiratory sinus arrhythmia (RSA), the degree of variability in heart rate associated with respiration, which provides a reasonable index of the activity of the vagus nerve referred to as ‘vagal tone’. Change of RSA in response to a stressor, termed ‘vagal withdrawal’, is thought to reflect an individual's capacity to regulate cognitive and emotional processes and therefore respond effectively to a challenge. Vagal withdrawal in children has been shown to protect them from the effects of adverse environments. For example higher vagal withdrawal buffered boys against increased externalizing symptoms related to exposure to more frequent marital conflict, especially verbal conflict [13]. Negative emotionality is a core component of infant temperament comprising anger proneness to constraints such as being placed in a car seat and fearfulness to unfamiliar events such as the approach of strangers [14]. Elevated anger proneness is associated with conduct disorders [15] and fearfulness with anxiety disorders [16] later in childhood.

Early postnatal experiences also have long term effects. In rats, maternal behaviors in the first days after birth have sustained effects on offspring stress reactivity and mothering of the next generation. These are explained by environmentally mediated effects of specific maternal behaviors: tactile stimulation, notably arch back nursing, and licking and grooming (LG) [17]. LG has been shown to lead to increased GR expression, enhanced HPA axis feedback regulation, and reduced CRF in brain structures involved in the hormonal, autonomic and emotional components of the stress reaction, and decreased fearfulness. A series of in vivo and in vitro studies has demonstrated that LG acts by stimulating expression and binding of transcription factors in the promoter region of the GR gene [18]. These are implicated in demethylation of key areas of the promoter region leading to stable, long term availability of the DNA segment for transcription. Taken together these findings suggest that fetal programming effects may be modifiable via postnatal experiences. This is supported in animal studies which find that long term effects of prenatal stress on hippocampal neurogenesis [19] and on offspring maternal behaviors [20] are modified by postnatal experiences such as handling or maternal care from non-stressed mothers.

In the light of this evidence, we predicted that human equivalents of rat LG should modify associations between an index of prenatal stress, prenatal depression, and subsequent physiological and behavioral outcomes in offspring. Thus far no studies in humans have identified candidates for maternal behaviours that may be the equivalent of rat LG. However, in rats the effects of LG can be mimicked by stroking the pups with a brush [6], consequently we asked mothers on two occasions over the first 9 weeks of their infants' lives how often they stroked the face, limbs and body of their babies. We predicted that, in humans, the effect of maternal prenatal depression on infant reactivity would be modified by tactile stimulation over the first weeks of life assessed by how often mothers reported stroking their babies. Modification would be evidenced in a statistical interaction between depression and frequency of stroking on both biological and behavioral indices of stress reactivity.

## Materials and Methods

### Ethics Statement

Ethical approval for the study was granted by the Cheshire North and West Research Ethics Committee on the 27th June 2006. The letter confirming ethical agreement for the study (reference number 05/Q1506/107) stated, ‘On behalf of the Committee, I am pleased to confirm a favourable ethical approval for the above research on the basis described in the application form, protocol and supporting document as revised.’ Participants gave written informed consent.

### Sample

Mothers and infants participated in the Wirral Child Health & Development Study, in which a larger (‘extensive’) general population sample was used to provide a stratified simple random subsample, the ‘intensive’ sample, and both are then followed in tandem. This two stage stratified design enables intensive measurement, such as that used in this study to assess vagal reactivity, while collection of other measures across the whole sample allows a weighting back of the findings from the intensive subsample to give general population estimates. The stratification variable, inter-partner psychological abuse reported by the women [21], was chosen for its known association with a variety of risk factors for early child development. For this study of prenatal depression the stratification variable was effective – the mean EPDS scores in the low vs high risk strata were 6.67 s.d. 3.97 vs 9.86 s.d. 4.80, Cohen's d = .68, p<.001 for comparison of transformed scores. The participants were identified from consecutive first time mothers who booked for antenatal care at 12 weeks gestation between 12/02/2007 and 29/10/2008. The booking clinic was administered by the Wirral University Teaching Hospital which was the sole provider of universal prenatal care on the Wirral Peninsula.. Socioeconomic conditions on the Wirral range between the deprived inner city and affluent suburbs, but with low numbers from ethnic minorities. The study was introduced to the women by clinic midwives who asked for their agreement to be approached by study research midwives when they attended for ultrasound scanning at 20 weeks gestation. After obtaining written informed consent the study midwives administered questionnaires and an interview in the clinic. The extensive sample of 1233 women (mean age 26.8, s.d. 5.8 years, range 18–51) pregnant with their first child was recruited at 20 weeks of pregnancy and subsequently had a live singleton baby. They were assessed again when the infants were 8.9 (s.d. 2.4) weeks (‘9 weeks’).

**Figure 1:**
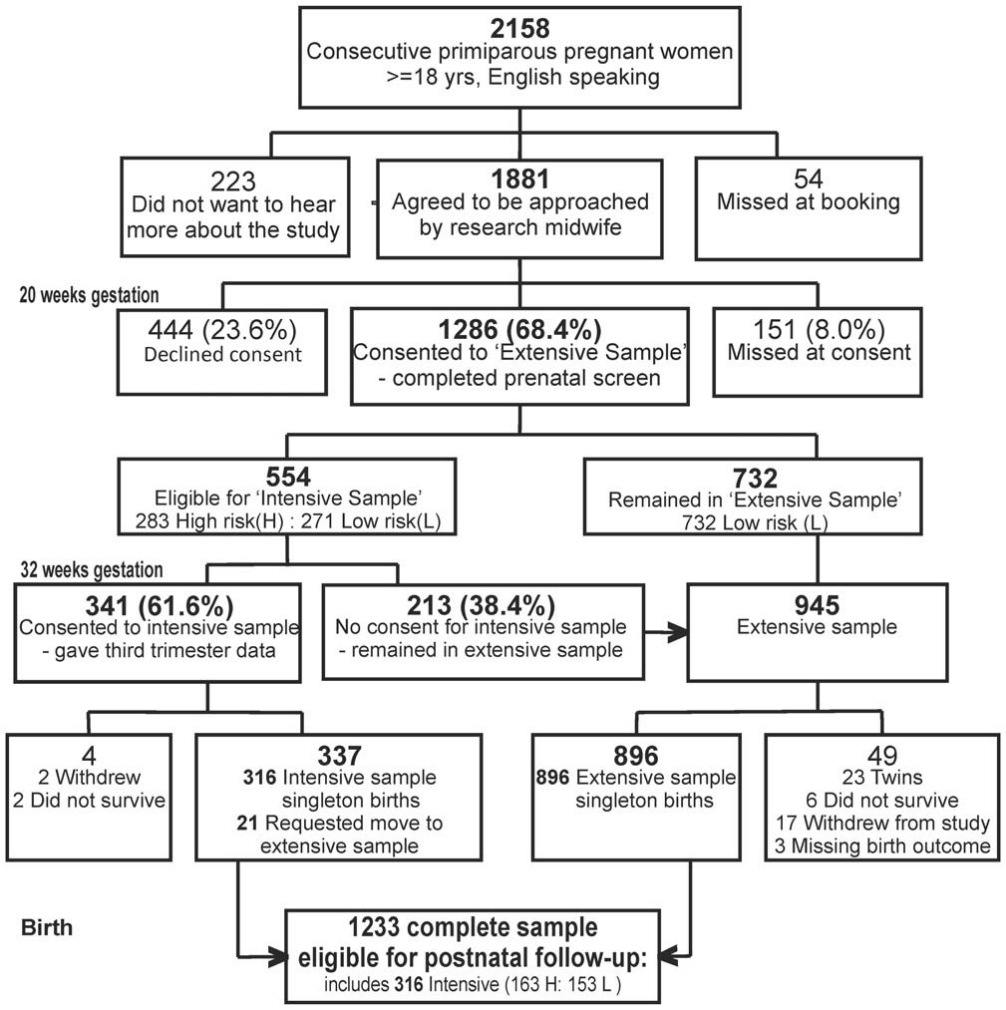
Sampling and recruitment.

All participants scoring above the threshold for psychological abuse towards themselves or their partners at 20 weeks gestation were eligible for inclusion in the intensive sample plus a random selection from those below the threshold. Within the intensively assessed stratified sub-sample, 51% were drawn from the women with high psychosocial risk and 49% from those with low psychosocial risk. The intensive sample of 316 women with surviving singleton births was assessed at 20 weeks of pregnancy (as part of the extensive sample), at mean 32.1 (s.d. 2.1) weeks of pregnancy (‘32 weeks prenatal’), and when the infants were mean 5.2 (s.d. 1.1) weeks (‘5 weeks’), 9 weeks (assessed as part of the extensive sample), and mean 29.1 (s.d. 3.1) weeks (‘29 weeks’) of age. The 271 mothers from the intensive subsample whose infants were assessed at 29 weeks were slightly older than the original extensive sample, mean age 27.9 years (s.d. 6.2, range 18–51). Recruitment to the sample is shown in Figure 1 and numbers and relevant measures at each assessment point in Table 1. Socioeconomic status was determined using the revised English Index of Multiple Deprivation (*IMD*) [22] based on data collected from the UK Census in 2001. According to this system, postcode areas in England are ranked from most deprived (i.e. IMD of 1) to least deprived (i.e. IMD of 32,482) based on deprivation in seven domains: income, employment, health, education and training, barriers to housing and services, living environment and crime. All mothers were given IMD ranks according to the postcode of the area where they lived and assigned to a quintile based on the UK distribution of deprivation. In the extensive sample 41.8% were in the most deprived UK quintile consistent with high levels of deprivation in some parts of the Wirral. A total of 48 women in the extensive sample (3.9%) described themselves as other than White British.

**Table 1:** Summary statistics of key measures.

	N	Mean	SD
**Extensive N = 1233 at 20 weeks gestation**			
Sex (female = 0,male = 1)	1233	0.49	0.50
Psychological Abuse, 20 weeks prenatal (Highest number of abusive behaviours from either partner to the other, max 20)	1233	0.86	1.87
Maternal report of Stroking frequency, 9 weeks post-natal (scored for each item from none = 1 to 5 = frequently)			
Tummy	866	3.80	0.91
Back	862	3.77	1.00
Face	865	4.12	0.80
Arms & legs	863	3.78	0.91
Breast feeding, refers to newborn & six weeks (scored none = 1 to 7 = mostly; mean across two time points)	850	4.28	2.46
Maternal Depression (EPDS 9 weeks, mean total score)	874	5.65	4.75
**Intensive N = 316 at 32 weeks gestation**			
Maternal Depression (EPDS 32 weeks prenatal; mean total score)	309	8.33	4.65
Maternal report of Stroking frequency, 5 weeks post-natal (scored for each item from none = 1 to 5 = frequently)			
Tummy	280	3.68	0.81
Back	280	3.79	0.99
Face	279	4.32	0.71
Arms & legs	279	3.77	0.87
Maternal Depression (EPDS 5 weeks; mean total score)	282	5.82	4.22
RSA 29 weeks post-natal – mean across each experimental procedure			
Helper hinderer task	270	3.18	0.85
New toy exploration	266	2.97	0.81
Face to face play with mother	257	3.32	0.84
Still face with mother	253	2.83	0.76
Resumption of face to face play with mother	247	3.35	0.95
Maternal rated infant distress (IBQ-R 29 weeks; mean item score)	252	3.26	0.84
Maternal rated infant fear (IBQ-R 29 weeks; mean item score)	251	2.17	0.82
Maternal Depression (EPDS 29 weeks; mean total score)	275	5.38	4.70

The estimate of Respiratory Sinus Arrhythmia (RSA) is derived by calculating the natural log of this variance and is reported in units of ln(ms)^2^. (28)

### Measures

#### Partner Psychological Abuse

Psychological abuse was assessed at 20 weeks pregnancy as humiliating, demeaning or threatening utterances in the partner relationship during the past year [21]. The scale is the total from 20 no-yes (coded as 0 absent, 1 present) items. Participants first rated these items about their own behavior towards their partner, and then about their partner's behavior towards them. This measure has been shown to yield large correlations between self and partner informant reports [21]. The psychological abuse variable used here was the highest of the partner to participant and participant to partner scores for each family.

#### Maternal Stroking

A literature review did not reveal any measures of infant stroking by mothers suitable for use within a large community sample. A brief self-report measure was devised (The Parent-Infant Caregiving Scale) in which mothers completed four items reporting on how often (1 = never, 2 = rarely, 3 = sometimes, 4 = often, 5 = a lot) they currently stroked their baby's face, back, tummy, arms and legs. This was completed on two separate occasions, at 5 and 9 weeks of age. Scores were derived as empirical Bayes' estimates from an ordinal item response model with different thresholds, scale and discrimination parameters for each item estimated in the gllamm procedure (http://www.gllamm.org) in Stata. The test-retest reliability over a 4 week period, between 5 week and 9 week stroking ratings was r = .58, p<.001. The robustness of the stroking effect was examined in analyses where the interaction between stroking assessed at 5 weeks and prenatal depression was replicated using the 9 weeks measure.

#### Maternal Depression

Maternal depression symptoms were assessed at 32 weeks prenatal and at 5, 9 and 29 weeks postnatal using the Edinburgh Postnatal Depression Scale [23].

#### Breast Feeding

As a test of the specificity of maternal stroking, we also examined effects of breastfeeding which also involves skin to skin contact, assessed by retrospective maternal reports at 9 weeks. Two items completed at the 9 weeks assessment asked about recalled use of breastfeeding when (a) the infant was newborn and (b) at six weeks of age. Mothers indicated the extent of breastfeeding on a 7 point likert scale with ‘mostly breast fed’ at one pole and ‘mostly bottle fed’ at the other. Scores were derived as the empirical Bayes' estimate from an ordinal logistic item response model with common thresholds, common factor variance, common scale parameter but different means over the two measurement occasions estimated in the gllamm procedure (http://www.gllamm.org).

#### Negative Emotionality

Negative emotionality was assessed using the ‘Distress to Limitations’ (anger proneness) and ‘Fear’ scales of the Infant Behavioral Questionnaire – Revised (IBQ – R) [14], based on maternal report. Distress to Limitations is a 16 item scale reflecting fussing, crying or showing distress while frustrated, for example, when put in a confined space. The Fear scale has 16 items assessing fear, startle or distress to strangers or novel stimuli. The IBQ-R has established reliability and validity and has been widely used in developmental studies [24], [25].

#### Respiratory Sinus Arrhythmia – Vagal Tone

Vagal tone was assessed as respiratory sinus arrhythmia (RSA). The experimental procedures for the assessment of vagal tone in this study were designed to address uncertainties in the research literature regarding the conditions for the measurement of baseline vagal tone, and hence of vagal withdrawal. According to Porges [26] baseline vagal tone is seen during quiet alert states, and in the absence of intense stimulation and concentration. Procedures for inducing these states have varied across studies, in some instances withdrawing all stimulation [27] with the risk that the child seeks attention and becomes distressed, and in others providing a standard low key stimulus [28]. In this study we used two low key conditions described below, the ‘helper-hinderer’ and ‘novel toy exploration’ to hold the infant's attention. We also addressed the question of whether there may be a latent vagal tone variable that represents a resting or baseline state and is reflected in RSA across all conditions whether or not they are challenging. This was examined using principal components analysis.

Respiratory sinus arrhythmia was computed from an ECG recording made during the five procedures described below. The recording was made from three Biopac (Biopac Systems, Inc., USA) pediatric disposable ECG electrodes placed on the infant's back connected to a Biopac Student MP35 acquisition unit box. The cardiac measurements were performed using the 3.9.1 version of the AcqKnowledge data recording software installed on a Windows XP laptop computer. The output was transmitted to the computer and stored for later off-line extraction of heart period data. All assessments were recorded on DVDs using a split-screen procedure, with 3 video outputs from the cameras and one showing the ECG trace. An electronic timer was also shown on the screen to code timings for procedures. Respiratory sinus arrhythmia was calculated by Cardioedit software using a procedure developed by Porges [29]. First, R-R (i.e. interbeat) intervals are timed to the nearest millisecond which results in a time series of consecutive heart periods (HP). Then an algorithm is applied to the sequential interbeat intervals data that uses a 3^rd^ order 21-point moving polynomial filter [30] which detrends periodicities in HP slower than RSA. A bandpass filter extracts the variance of HP within the frequency band of spontaneous respiration in infants (i.e. 0.24–1.04 Hz). Finally, RSA is derived by calculating the natural log of this variance and is reported in units of ln(msec).

*Procedure 1, The Helper-Hinderer* is an experimental paradigm developed to assess whether infants favour prosocial acts [31]. The infant is seated on the mother's lap and views a large display (3×5 feet) situated in front of him/her approximately 6 feet away in which a coloured shape (square, circle, triangle) with googly eyes is shown either helping another up a slope (helper trial) or hindering another's progress up the slope (hinderer trial). Helping trials and hindering trials are alternated throughout and the series of learning trials are ended once the infant has shown a predetermined level of habituation to the stimuli, or when the maximum number of pre-set trials has been reached (14 trials). Criterion for habituation was defined on an a priori basis as the point when the mean infant looking time over three consecutive trials had fallen to half the mean looking time observed over the first three trials. Once the learning trials ended the infant was given a preference task, between the helper-shape or hinderer-shape. Shape and colour of the stimuli are counterbalanced across trials. The duration of the learning procedure is not standard but varies depending on how quickly the infant habituates to the presentation of the stimuli. In the current study the mean duration of the procedure was 3.74 minutes, *SD* 1.20, minimum 0.88 minutes, maximum 8.09 minutes RSA was calculated for the last 2 minutes of this procedure to ensure standardisation of infants' looking times.

*Procedure 2, The Novel Toy Exploration Procedure* is a 2-minute episode in which the infant is presented at a table with a 4-facet triangular pyramid-shaped toy to explore for two minutes while sitting on mother's knees. This has been used in previous studies to assess baseline vagal tone [32].

*Procedures 3, 4, and 5, The Still-face procedure* was conducted with the infant in a high chair facing the mother. This comprised two minutes of face to face playful interactions without toys, followed by two minutes during which the mother was asked to be unresponsive to her child's communications (the ‘still face’), followed by two minutes during which she was asked to become responsive again (the ‘repair’) [33]. The Still Face has been used extensively in studies of vagal reactivity to stress [27], [34].

The numbers of infants from whom RSA data were obtained varied between 270 and 247 by condition (Table 1) because the electrodes became detached in 26 infants at various points during the procedures. Principal components analysis yielded a factor with an Eigenvalue of 3.54 which explained 70.73% of the total variance. All five RSA values loaded highly on to the factor (factor loadings .86, .84, .84, .84, .82) supporting the existence of a latent variable which could be construed as ‘resting’ or ‘baseline’. For simpler analyses and for graphical description of effects, baseline vagal tone was measured by the average RSA across the helper-hinderer, novel toy, engagement and repair conditions. Vagal withdrawal was measured as this average minus the RSA under the still-face condition.

### Statistical Analyses

A joint analysis of baseline vagal tone, vagal withdrawal and Infant Behavior Questionnaire (IBQ) distress to limitations and fear, each normally distributed, was undertaken using multivariate regression. As shown in Table 2 estimates of the primary effects of interest - prenatal depression, maternal stroking, and their interaction - were obtained both before and after inclusion of potential confounders (maternal depression at 5, 9, and 29 weeks and breast-feeding). Breast feeding was included as a predictor of each outcome both as a main effect and in interaction with the index of prenatal stress, maternal depression. The stratification variable was also included as a covariate to verify that the estimated effects did not arise from sample selection bias. We report analyses for all infants with RSA following multiple imputation for occasional missing covariates (100 replicates). Multiple imputation was carried using the ice procedure in Stata [35], [36] allowing for all the main effects and interactions that formed part of the analysis. All tests reported were two-tailed Wald tests.

**Table 2:** Summary of multivariate regression analyses showing coefficients (standard errors) and significance for the effect of maternal report of stroking and prenatal depression with adjustment for sample stratification and 5, 9 and 29 week postnatal depression and breast feeding confounders.

*Standardized Effect*	*Baseline Vagal tone*		*Vagal withdrawal*		*IBQ Distress to Limitations*		*IBQ Fear*	
Simple	Coeff.(SE)	P	Coeff.(SE)	P	Coeff.(SE)	P	Coeff.(SE)	P
**Model**								
Prenatal Dep. (D)	.015(.06)	.80	.032(.06)	.62	.153(.06)	.015	.057(.06)	.34
Stroking (S)	.031(.12)	.76	−.259(.12)	.03	.203(.12)	.09	.160(.12)	.18
**D×S int.**	**−.020(.12)**	**.80**	**.314(.12)**	**.01**	**−.321(.12)**	**.007**	**−.243(.12)**	**.043**
**Model with confounders^a^ & stratifier^b^**								
Prenatal Dep. (D)	.069(.08)	.42	−.025(.09)	.78	−.013(.09)	.88	−.063(.09)	.47
Stroking (S)	.068(.12)	.57	−.266(.12)	.03	.180(.12)	.13	.166(.12)	.17
**D×S int.**	**−.067(.12)**	**.58**	**.323(.12)**	**.008**	**−.293(.12)**	**.016**	**−.229(.12)**	**.06**
Maternal Dep.	−.155(.09)	.10	.021(.10)	.83	.070(.01)	.47	.103(.10)	.30
5 weeks								
Maternal Dep.	.034(.11)	.76	−.168(.12)	.15	.009(.12)	.94	−.098(.12)	.41
9 weeks								
Maternal Dep. 29weeks^a^	−.018(.08)	.83	.120(.09)	.18	.166(.09)	.05	.139(.09)	.12
Stratifier. ^b^	−.023(.06)	.74	.033(.07)	.64	.041(.07)	.56	.073(.07)	.31
Breastfeed^a^ (B)	.096(.14)	.49	.143(.14)	.32	.077(.14)	.58	.202(.15)	.04
**D×B int.^a^**	**.041(.14)**	**.76**	**−.015(.14)**	**.92**	**−.172(.14)**	**.21**	**−.189(.14)**	**.19**

The main and interaction effects (the latter in bold) are shown of prenatal depression and breast feeding with maternal report of stroking on vagal withdrawal and infant negative emotionality – distress to limitations and fear - at 29 weeks. Estimates from 100 multiple imputation replicates with sample N = 271.

**Figure 2:**
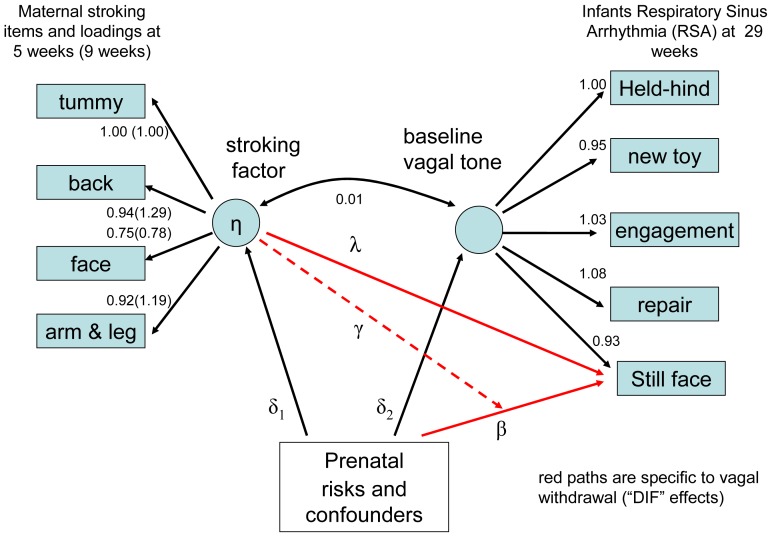
Latent variable model for stroking items and respiratory sinus arrhythmia (RSA) estimate of vagal tone. The figure shows the factor loadings for mothers' reports of stroking at 5 and 9 weeks, and RSA at 29 weeks. The values of λ (direct effect of stroking on vagal withdrawal), δ_1_, δ_2_, and β (associations of risks and confounders with stroking, vagal tone and vagal withdrawal respectively), and γ (interaction between maternal stroking and prenatal maternal depression), are shown in Table 3.

**Figure 3:**
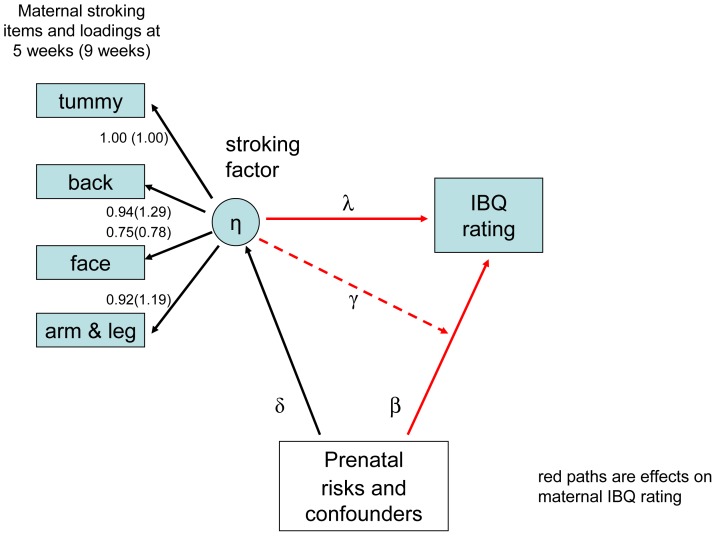
Latent variable model for stroking items and IBQ negative emotionality. The figure shows the factor loadings for mothers' reports of stroking at 5 and 9 weeks, and IBQ negative emotionality (Distress to Limitations and Fear) at 29 weeks. The values of λ (direct effect of stroking on IBQ), δ and β (associations of risks and confounders with stroking and IBQ respectively), and γ (interaction between maternal stroking and prenatal maternal depression), are shown in Table 4.

As a further check on the robustness of findings we fitted by maximum-likelihood a latent variable model using gllamm shown in Figure 2 for vagal tone and in Figure 3 for negative emotionality, using data from the general population extensive and stratified intensive samples. The 5 weeks and 9 weeks stroking data were analysed separately for computational simplicity and to test for replication with this novel measure. The left of the diagram shows the one-factor item-response measurement model fitted to the four 5-category ordinal stroking items (each item has 4 threshold parameters and a factor loading (discrimination parameter)). The stroking factor may be correlated with the baseline vagal tone factor but may also have a direct impact on vagal withdrawal (through λ). In addition, as illustrated for a single risk factor, risks and confounders may be associated with stroking (through δ_1_) and baseline vagal tone (through δ_2_) and may influence vagal withdrawal (through β) directly. Finally, the impact of the prenatal risk may be moderated by the stroking score, giving an interaction effect (through γ). Algebraically, the total direct effect on vagal withdrawal for infant j is given by λη_j_+βx_j_+γx_j_η_j_, where x is the prenatal risk and η is the stroking factor and the γ parameters estimate the interaction effect of measured prenatal risk and latent stroking.

## Results

The analysis is based on the 271 infants (of the 316 in the intensive sample) for whom a baseline vagal tone at 29 weeks of age could be estimated. Table 1 shows descriptive statistics for the extensive and intensive samples prior to imputation. Prenatal maternal depression (32 weeks gestation) was significantly correlated with infant distress to limitations at 29 weeks (r = .16, p = .01), but not with vagal tone (r = .02, p = .80), vagal withdrawal (r = .03, p = .66), or infant fearfulness (r = .08, p = .22). The measure of baby stroking proved to be only weakly correlated with prenatal risk factors such as 32-week prenatal depression (r = −.06; p = .34), male infant (r = −.01, p = .77), early pregnancy psychological abuse (r = .01, p = .85), single parenthood (r = −.03, p = .85 and postnatal depression at 5 weeks (r = −.09, p = .12), 9 weeks (r = −.03) and 29 weeks (r = .00, p = .99), and breast feeding (r = .03, p = .39). The direct associations of maternal stroking with the four outcomes were also low; baseline vagal tone (r = .01, p = .86), vagal withdrawal (r = .01, p = .92) and maternally reported distress to limitations (r = −.08, p = .23) and fear (r = −.04, p = .53).

**Figure 4:**
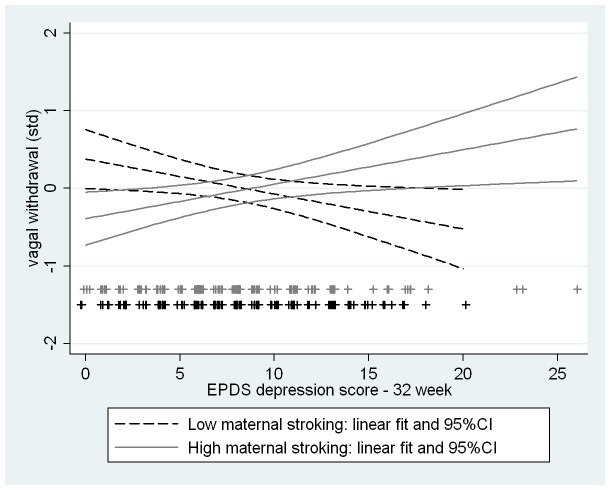
Interaction between maternal stroking and prenatal depression on infant vagal withdrawal. Simple regression lines and 95% confidence envelopes showing the interaction between maternal reports of stroking (median split) and prenatal depression, with infant vagal withdrawal at 29 weeks (p = 0.01 from multivariate regression).

**Figure 5:**
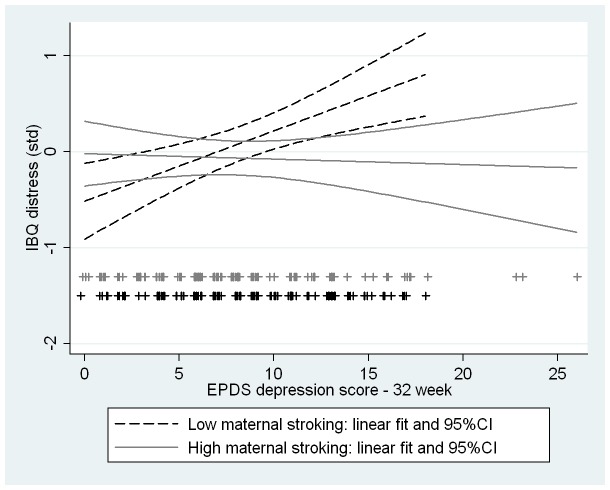
Interaction between maternal stroking and prenatal depression on infant distress to limitations. Simple regression lines and 95% confidence envelopes showing the interaction between maternal reports of stroking (median split) and prenatal depression, with infant IBQ distress to limitations at 29 weeks (p = 0.007 from multivariate regression).

The first three rows of Table 2 show for each of the outcomes the standardized estimates for the main effects and the interaction (product) effect of prenatal depression score and stroking. While the interaction of prenatal depression and maternal stroking on the reference baseline indicator of vagal tone was small and wholly non-significant, those on the outcomes assessing vagal withdrawal and behavioral reactivity were significant. The effects both on vagal withdrawal (b = .31; 95% CI 0.08, 0.55) and maternal-rated infant distress to limitations (b = −.32; 95% CI −.55, −.09) were large and that for fear was more modest but still significant (b = −.24; 95% CI −.48,−.01). As shown in the lower part of Table 2, the analyses were repeated with the addition of controls for sample stratification and confounders, notably post-natal depression and breastfeeding, and the effects were very similar although that on infant fear was no longer statistically significant. Figures 4 and 5 show these interaction effects among the complete unimputed data cases (N = 225) showing the regression lines of high and low maternal stroking groups when divided at the median. In the low stroking group prenatal maternal depression was associated with decreasing vagal withdrawal (variance explained = 4%, standardized coefficient = −.20, p = .037) but the association was in the opposite direction in the infants of high stroking mothers (variance explained = 4%, standardized coefficient = .21, p = .013). Prenatal maternal depression was also associated with increasing distress to limitations and fear, but only in the infants of mothers below the median for stroking. In the low stroking group prenatal depression explained 9% of the variance in distress to limitations (standardized coefficient = .33, p = .001) and 4% of the variance in fear (standardized coefficient = .21, p = .033). In the high stroking group prenatal depression predicted neither distress to limitations (p = .89) nor fear (p = .47). By contrast interactions between prenatal depression and breastfeeding were all non-significant. Post-hoc power analysis using complete data cases and simple regression (powerreg procedure in Stata) gave 72% power for the interaction with vagal withdrawal and 81% power for the interaction with distress to limitations.

Parameter estimates of primary scientific interest for models for vagal withdrawal in which postnatal depression and breastfeeding are potential confounders and the sample design is accounted for by inclusion of the stratification variable from fitting a maximum-likelihood latent variable model are shown in Table 3. For the analysis with stroking at 5 weeks the only significant effect of interest is the interaction of latent stroking and pre-natal depression on vagal withdrawal – the γ coefficient (likelihood-ratio χ^2^ p = .026). The same interaction also appeared significant using the 9 week measure of stroking (likelihood-ratio χ^2^ p = .046). Essentially the same model was estimated to examine the effects on maternal ratings of infant distress to limitations and fear with estimates shown in Table 4. With the 5-week measure of stroking again we found that the only significant effect was the interaction of prenatal depression and the latent variable stroking factor on both distress to limitations and fear. As with vagal withdrawal, the estimated effects with the 9 week stroking measure were similar or larger but were less well estimated and not quite significant.

**Table 3:** Parameter Estimates and Standard Errors for the Vagal Tone (RSA) Model of Figure S2 (which shows the stroking and vagal tone factor loadings) for the effects of stroking with adjustment for stratification and confounders.

Risk factors Parameter	Effect estimates for model with stroking at 5 weeks and with confounders and stratifier	Effect estimates for model with stroking at 9 weeks and with confounders and stratifier
**λ stroking on still face**	−.016 (.015)	.008 (.029)
**δ_1_ prenatal depression on stroking**		
32-wk prenatal depression	−.126 (.249)	.091 (.157)
Stratifier^a^	−.113 (.147	−.012 (.094)
5-wk depression	−.150 (.268)	−.295 (.171)
29-wk depression	.289 (.259)	.115 (.158)
Breast feeding	−.001 (.198)	−.154 (.125)
**δ_2_ prenatal depression on**		
**vagal tone**		
32-wk prenatal depression	.015 (.065)	.015 (.065)
Stratifier^a^	.005 (.038)	.005 (.038)
5-wk depression	−.038 (.069)	−.038 (.069)
29-wk depression	−.047 (.065)	−.046 (.065)
Breast feeding	.117 (.052)	.116 (.051)
**β prenatal depression on**		
**vagal withdrawal**		
32-wk prenatal depression	−.010 (.047)	−.011 (.047)
Stratifier^a^	−.028 (.027)	−.030 (.027)
5-wk depression	.114 (.052)	.108 (.051)
29-wk depression	−.095 (.051)	−.085 (.050)
Breast feeding	−.075 (.037)	−.058 (.037)
**γ maternal stroking by prenatal**		
**depression interaction on vagal**	**−.035 (.014)**	**−.061 (.027)**
**withdrawal**	**LR p = .009**	**LR p = .020**

**Table 4:** Parameter Estimates and Standard Errors for the IBQ Distress to Limitations and IBQ Fear model of Figure S3 (which shows the stroking factor loadings) for the effects of stroking with adjustment for stratification and confounders.

Risk factors	Effect estimates for model with stroking at 5weeks and with confounders and stratifier		Effect estimates for model with stroking at 9 weeks and with confounders and stratifier	
Parameter	Distress	Fear	Distress	Fear
**λ stroking on IBQ**	−.014 (.023)	.002 (.023)	−.070 (.044)	−.021 (.043)
**δ prenatal depression on**				
**stroking**				
32-wk prenatal depression	−.197 (.251)	−.176 (.250)	.062 (.160)	.070 (.160)
Stratifier^a^	−.105(.147)	−.101 (.147)	−.000 (.095)	−.003 (.095)
5-wk depression	−.163 (.269)	−.210 (.267)	−.306 (.173)	−.320 (.173)
29 wk depression	.336 (.256)	.390 (.255)	.142 (.159)	.164 (.159)
Breast feeding	−.034 (.199)	−.045 (.199)	−.163 (.127)	−.174 (.127)
**β prenatal depression**			.	
**on IBQ**				
32-wk prenatal depression	.077 (.074)	−.004 (.072)	.074 (.075)	−.007 (.072)
Stratifier^a^	.015 (.044)	.059 (.042)	−.003 (.044)	.051 (.042)
5-wk depression	.046 (.079)	−.008 (.076)	.039 (.080)	−.014 (.077)
29-wk depression	.129 (.075)	.118 (.072)	.133 (.075)	.121 (.073)
Breast feeding	−.060 (.058)	.083 (.056)	−.037 (.059)	.104 (.057)
**γ maternal stroking by**	**−.052 (.021)**	**−.050 (.020)**	**−.070 (.042)**	**−.070 (.040)**
**prenatal depression**	**LR p = .013**	**LR p = .014**	**LR p = .091**	**LR p = .081**
**interaction on IBQ**				

## Discussion

Frequency of infant stroking, assessed via maternal self-report at two time points in the early postnatal period, modified associations between prenatal maternal depression and both infant physiology and emotional reactivity. The effect of prenatal depression on the infant outcomes differed depending on post-natal exposure to maternal stroking, as evidenced in a statistical interaction between prenatal depression and maternal stroking. In each case the direction of effects was the same. Increasing maternal depression was associated with decreasing vagal withdrawal, a measure of physiological adaptability, and with increasing negative emotionality, only in the presence of low maternal stroking. These findings represent initial evidence in humans of maternal behaviors that modify developmental outcomes of prenatal stress in a manner analogous to the effects of early maternal behaviors on gene expression and stress reactivity seen in rodents.

We used maternal report of stroking as it draws on behaviour that spans contexts in a way that experimental or naturalistic observation of a large community sample could not. The measure was devised for this study because no previous suitable measures could be identified in the literature. The four stroking items clearly assessed a stroking construct as evidenced in high loadings of all of the items on a latent variable in the models shown in Figures 2 and [3]. Test retest reliability was good and similar to that of maternal reports of infant temperament [24]. The construct validity of the stroking measure was supported by findings, over six analyses, each consistent with translation from the animal work. Using the measure we first showed interactions of maternal report of stroking with prenatal depression, in the same direction, for the prediction of autonomic reactivity, anger proneness and fearfulness, and then in independent analyses of reports of stroking when the infants were 5 weeks and again at 9 weeks of age. Further support for construct validity of the stroking measure will require additional findings consistent with predictions based on the biology of early development, within this and other samples. Discriminant validity was supported by the finding that breast feeding, which also entails skin to skin contact, did not predict the physiological and behavioural outcomes. In the future demonstrating agreement with observational measures will also be relevant to establishing validity although such observational measures are generally limited in studies of human development by restricted coverage over place and time, and so cannot straightforwardly be considered as ‘gold standard’. As in the case of temperament research in infancy, in the absence of an agreed gold standard, self-report and observational measures perform complementary functions and so the further investigation of maternal stroking in infancy is likely to be approached similarly [14]. In this study we did seek to mitigate the risks of bias associated with self-report by controlling for maternal mood assessed when the questionnaires were administered.

Extrapolation from maternal care in rats to humans may seem unwarranted given the complexity of human parenting and infant development. In particular, compared to other mammals, primates have vastly greater and more complex social competences, and humans more than non-human primates [37]. Variations in well established dimensions of parenting, such as sensitivity, intrusiveness, or hostility with effects on attachment security, emotionality and social attributions may be expected to be the most salient for long term effects [38]. Equally, however, long term parental effects on multiple physiological systems, are common in biology, and likely to be mediated via epigenetic modifications [39],[40]. These may represent an evolved capability for environmentally sensitive plasticity over a limited number of generations, which could be particularly important in species, such as humans, with lengthy time periods between generations and hence only slow genotypic selection in relation to changing environmental conditions [6]. Importantly, tactile stimulation derived from parental care has immediate effects on endocrine systems that regulate somatic growth in rodents [41],[42] and humans [43] suggesting that the ability of the infant to respond to specific forms of parental care is conserved at least among mammals. In the context of the current findings, the genetic mechanisms in glucocorticoid and CRF regulation are highly conserved across species [44], and so an effect of tactile stimulation on GR expression may have been conserved across rodents and humans.

In common with many other studies of the developmental consequences of prenatal affective symptoms we used a self-report measure of symptoms of depression. It remains to be seen whether maternal stroking also modifies outcomes following diagnosed depressive disorder assessed by interview. These findings need replication in other longitudinal studies and naturalistic observational methods of assessing parental stroking need to be established. Frequency of parental stroking may vary under different environmental conditions, and so future experimental observational studies might contrast stroking frequencies under standardized stressful and non-stressful conditions. Many other questions remain to be addressed. For example, although we could find no evidence that maternal stroking was a function of a mother's level of depression either before or after the birth of her child, nor that the effect could be explained by the extent of another maternal behavior that includes skin to skin contact, namely breast feeding, it remains a distinct possibility that stroking is only a proxy for another causal aspect of parenting. To further substantiate this as the counterpart of the maternal tactile stimulation mechanism seen in rats, effects of experimentally generated, as well as naturally occurring, variations in stroking could be explored. Examination of gene expression in humans is currently limited to peripheral tissue, however against the background of extensive animal work, demonstrating differences in GR expression within experimental designs would be highly informative.
